# Low-Frequency Pulsed Electromagnetic Field Is Able to Modulate miRNAs in an Experimental Cell Model of Alzheimer's Disease

**DOI:** 10.1155/2017/2530270

**Published:** 2017-05-02

**Authors:** Enrica Capelli, Filippo Torrisi, Letizia Venturini, Maria Granato, Lorenzo Fassina, Giuseppe Francesco Damiano Lupo, Giovanni Ricevuti

**Affiliations:** ^1^Department of Earth and Environmental Sciences, Section of Animal Biology, University of Pavia, Via Taramelli 24, 27100 Pavia, Italy; ^2^Centre for Health Technologies (C.H.T.), University of Pavia, Via Ferrata 1, 27100 Pavia, Italy; ^3^Department of Internal Medicine and Therapeutics, Section of Geriatrics and Gerontology, IDR “Santa Margherita”, University of Pavia, Via Emilia 12, 27100 Pavia, Italy; ^4^Department of Electrical, Computer and Biomedical Engineering, University of Pavia, Via Ferrata 1, 27100 Pavia, Italy

## Abstract

The aim of the present study was to investigate on the effects of a low-frequency pulsed electromagnetic field (LF-PEMF) in an experimental cell model of Alzheimer's disease (AD) to assess new therapies that counteract neurodegeneration. In recent scientific literature, it is documented that the deep brain stimulation via electromagnetic fields (EMFs) modulates the neurophysiological activity of the pathological circuits and produces clinical benefits in AD patients. EMFs are applied for tissue regeneration because of their ability to stimulate cell proliferation and immune functions via the HSP70 protein family. However, the effects of EMFs are still controversial and further investigations are required. Our results demonstrate the ability of our LF-PEMF to modulate gene expression in cell functions that are dysregulated in AD (i.e., BACE1) and that these effects can be modulated with different treatment conditions. Of relevance, we will focus on miRNAs regulating the pathways involved in brain degenerative disorders.

## 1. Introduction

Alzheimer's disease (AD) is a neurodegenerative disorder with irreversible progression that primarily affects the hippocampal and neocortical regions of the brain. Since the incidence of AD increases in the elderly and with the lengthening of human life, this disease is becoming one of the major health problems associated with aging [1]. There is currently no effective treatment against AD, and its pathogenesis remains unclear [2]. A lot of studies on AD have highlighted the possible involvement of genetic [3], immunological [4], and environmental causes [5]. Oxidative stress, disruption of calcium homeostasis, hormonal factors, inflammation, and vascular and cell cycle dysregulations have been associated with the disease [6]. The major microscopic abnormalities of AD, which form the basis of the histologic diagnosis, are *β*-amyloid (A*β*) plaques and neurofibrillary degeneration (tangles). Notably, the neuritic plaques are mainly composed of A*β* secreted through an aberrant proteolytic cleavage of the amyloid precursor protein (APP) [7]. There are progressive and eventually severe neuronal loss, synaptic loss, and reactive gliosis in the same regions that bear the burden of the plaques and tangles. The involvement of the hippocampus and amygdale in the early phases of AD causes synaptic dysfunctions, such as the block of long-term potentiation (LTP), with consequent damage of the processes of learning and memory [8].

On the other hand, a new study about the brain and the electromagnetic fields (EMFs) showed, in vivo, that the EMFs could protect from the cognitive impairment or improve the memory in mice [9], whereas other in vitro studies indicated a possible role of EMFs as copromoters of tumor growth [10].

Besides age, family history, and inheritance, which are considered important risk factors, emerging evidence suggests that also environmental factors can influence AD development and progression, especially with regard to the sporadic disease which represents the most widespread form. In general, the physiopathological conditions within cells, tissues, and organs can be influenced by changes in the electromagnetic context to the extent that even their phenotype and functions can be altered by EMF exposure [11, 12]. Some literature data indicate that EMFs seem to play a role in the etiology of neurodegenerative disorders, including AD [13, 14]. Interestingly, although the debate on EMFs is still controversial, a pioneering field of research in AD is the deep brain stimulation via EMFs, which seems to modulate the neurophysiological activity of the pathological circuits and produce clinical benefits in AD patients [15]. Of relevance, in recent years, EMF brain stimulation techniques, such as the transcranial magnetic stimulation (TMS) (which noninvasively interacts with the brain activity), have been developed and applied to treat neurological diseases. TMS-induced cortical changes have resulted in enhanced neural plasticity. Indeed, an enhancement of the brain cortical excitability might induce a specific potentiation-like phenomenon, which would enable synaptic plasticity and promote recovery of a degraded function. Given these premises, there is currently a growing interest in applying EMFs as a therapeutic approach in psichiatric and neurological disorders [16]. Moreover, EMFs could be clinically used to re-establish cognitive performance in stroke patients [17, 18] and in patients suffering from neurodegenerative diseases [19, 20]. Presently, various clinical trials are ongoing to further investigate the possible positive effects of EMFs and TMS on AD (www.clinicaltrials.gov).

Despite the significant use of brain stimulation in clinical treatments, as mentioned, the effects of EMFs on the biological systems are not completely understood. In fact, it has been observed that, depending on the EMFs' “dose” and wavelength, the effects can shift from cytotoxicity to cytoprotection [21–23]. As recently reported [24], the electromagnetic waves are able to modulate the cytoskeleton function and to promote the neuronal differentiation of the bone marrow mesenchymal stem cells; in particular, EMFs promote the neuronal differentiation in vitro and the hippocampal neurogenesis in vivo by upregulating the Cav-1 channel activity [25–28], *β*-III-tubulin, MAP2 [29], and the brain-derived neurotrophic factor [30].

At a molecular level, it has been postulated that EMFs can affect the redox status within cells, thus evoking a general stress response [31] and increasing the expression of stress-related proteins [32]. Moreover, it has been reported that EMFs can delay cellular senescence [33]. As previously shown on an AD mice model, a high-frequency EMF treatment induced an improvement of cognitive functions, ascribed to an enhanced clearance of the amyloid plaques [9]. Conversely, in an in vitro cellular AD model overexpressing APP, prolonged EMFs caused a significantly increased secretion of A*β*_1–42_ [34], one of the most prone-to-aggregation APP derived fragments [7].

Of interest, it has been widely demonstrated, in vitro, that both low- and high-frequency EMFs can also modulate gene expression by acting on both transcriptional and posttranscriptional regulatory mechanisms [35–37]. Within this context, in both physiological and pathological conditions, posttranscriptional mechanisms are key determinants of the gene expression modulation, since they allow a rapid adaptation of protein levels to changing environmental conditions and can differently influence the cell fate. These mechanisms include the implication of a class of small noncoding RNA molecules, called miRNAs, able to regulate the gene expression mainly by base pairing to the 3′-UTR of specific target mRNAs [38]. Considering that miRNAs are predicted to regulate up to 90% of human genes [39], their physiological activity is critical for the maintenance of healthy conditions and their aberrant expression is associated with the pathological features of many diseases [38, 40].

In particular, mRNA is ~5% of the total cellular RNA and is poorly correlated with protein levels. It is increasingly clear that mRNA translation is a key focal point of gene expression regulation. Noteworthy for this project, miRNAs regulate the expression of key proteins involved in AD pathogenesis and the expression of certain miRNAs is altered in AD patients [41–45], thus suggesting that a dysfunctional miRNA-based regulatory system may represent a new etiologic factor for AD. Notably, an alteration of several miRNAs has been related to A*β* insult [46]. Many other miRNAs are emerging as regulators of the expression not only of APP but also of proteins involved in fundamental cellular processes such as cellular clearance and quality control systems which are altered in AD [47]. Recently, it has been suggested that miRNAs are also able to modulate cognitive and immune processes through direct or indirect alterations of the neuron-to-glia and/or the brain-to-body signaling [48]. In line with this concept, gene expression studies on AD and control subjects have shown differences in some miRNAs not only in the affected brain areas and in the cerebral spinal fluid but also in the peripheral districts, such as blood [49]. Very recently, the potential contribution of miRNAs to AD pathophysiology in humans and in various cellular and animal models has been remarked [50]. Furthermore, a lot of studies have documented the presence of miRNAs (and other RNAs) in the extracellular space after their release from the cells and in the circulating blood. These miRNAs are contained within a variety of different structures and protein/lipoprotein complexes [51, 52]. The circulating miRNAs appear to escape degradation via endogenous ribonuclease activity by residing in membrane-structured bodies as well as protein and lipid complexes [53]. miRNAs previously move through the bloodstream from one district to the others [54, 55]. Circulating miRNAs have emerged as candidate biomarkers for a long list of diseases and medical conditions [56]. Therefore, miRNAs may represent a fine-tuning of the signaling able to reach different body districts and able to integrate multiple inputs and outputs [57]. In this scenario, a deeper understanding of the relation between AD, EMFs, and miRNAs may help to shed more light on the molecular bases of this pathology, also opening the possibility towards the use of miRNAs as potential clinical biomarkers. For instance, it has been demonstrated that the transcranial electromagnetic stimulation of the brain through pulsed electromagnetic fields (PEMFs) can establish the reactivation of cognitive processes in AD patients and the reduction of A*β* in transgenic mice models for APP [9].

Considering these preconditions, the relationships between low-frequency PEMF (LF-PEMF) exposure and miRNAs regulating the proteins connected with altered functions in AD might explain the molecular basis of neuropathologies and show new therapies. miRNAs could be used as drugs to block the production of harmful proteins in new therapeutic strategies, because of their capacity to downregulate gene expression up to silencing, through the interaction with their target messengers. It is important to identify miRNAs that are modulated by exposure to LF-PEMFs in order to characterize the mechanisms associated with AD. Finally, since there are conflicting data about the effects of the electromagnetic fields and various publications deal toxic actions, further studies on LF-PEMFs' effects are necessary to verify whether the exposure to certain dosages may induce therapeutic advantages or, on the contrary, constitute an additional risk factor.

As a consequence, the aim of our study was to evaluate the modulation of miRNAs induced by LF-PEMF in the peripheral blood mononuclear cells (PBMCs) obtained from AD patients. PBMC exposure was realized using an electromagnetic bioreactor, with a frequency of 75 Hz [58]. Significant miRNAs were selected following a search in miRBase, TarBase, and miRTarBase databases. hsa-miR-107 regulates the enzyme BACE1, which exerts its action determining the amyloidogenic pathway of APP protein. Previous research identified a reduced expression of miRNA 107 in AD patients; since this miRNA negatively regulates BACE1, its lower expression promotes the production of toxic peptides A*β*_40_ and/or A*β*_42_. Then, we decided to check whether the treatment with LF-PEMF leads to an increased expression of miRNA 107 and, so, to a lower production of toxic peptides of A*β*, achieving a clinical benefit.

Moreover, we considered other significant miRNAs such as hsa-miR-335-5p that targets the MAPK1 gene, which encodes for one of the extracellular signal-regulated kinase (ERK) proteins, a mitogen-activated protein involved in cell growth and in the long-term potentiation (LTP) and acting in synapses regeneration. The same miRNA targets the GRIA1 (glutamate ionotropic receptor AMPA type subunit 1) gene, encoding for the AMPA receptor 1, which is essential for the first phase of LTP induction. Consequently, after LF-PEMF stimulation, a low expression of miR-335, which determines an increase of ERK and AMPA receptor, may be positive for both the cell regeneration and the neurological processes that regulate memory and learning. hsa-miR-26b-5p regulates the expression of the SLC17A6 gene, which encodes for the transporter vGLUT_2_. This transporter puts glutamate in presynaptic vesicles, which will be released to reach the postsynaptic terminal, where they can interact with AMPA and NMDA receptors. So, we decided to determine whether the action of the LF-PEMF can modulate the expression of this miRNA, since a possible increased vGLUT_2_ level may cause a higher intake of glutamate within the presynaptic vesicles. This protects the nervous system from the excitotoxicity of the glutamate itself and triggers LTP processes, improving memory and cognition.

### 1.1. Electromagnetic Fields and ROS in Alzheimer's Disease

At the molecular level, PEMFs have been hypothesized to affect the redox status of the cells, causing protein stress [32]. Also, antioxidant activity is modulated by PEMFs. A stimulation of the antioxidant activity, demonstrated by a decrease of 58.31% of the average in malondialdehyde value and by the balancing of the redox status, was observed in healthy volunteers [59]. The balance between the free radicals and antioxidants (redox equilibrium) is a critical point for the maintenance of homeostasis in a biological system: reactive oxygen species (ROS) at high doses are deleterious because they cause pathophysiological actions, whereas at low doses, they may be beneficial for normal physiological functions such as signal transduction, gene expression, and regulation of the immune response and for the strengthening of antioxidant defense mechanisms [60].

During the experiments on PBMCs of AD patients, electromagnetic waves have been observed to cause a growth of the total production of ROS; this increase seems to be linked to the timing of exposure [61]. The stimulus applied is able to primarily determine a strong increase of ROS until reaching a plateau and then, a decrease with the time. An initial increase, linked to the timing applied, suggests a ROS-mediated amplification of the inflammatory response [62]. The same trend is observed in cultured neurons treated with A*β*, suggesting the role of EMFs in the further activation of the cells defending the tissue damaged by A*β*. A ROS increase could also be responsible for an increase of autophagy and “phagocytic clearance” by microglia which can eliminate the A*β*. The increase of ROS could acquire the role of a “priming agent” as being responsible for the creation of a preconditioning aimed at the clearance of potentially hazardous substances [63]. So, the cognitive improvement and the reduction of A*β* plaques, after electromagnetic fields stimulation, may depend primarily on the enhancement of ROS-mediated inflammatory response after exposure.

### 1.2. Electromagnetic Fields and Synaptic Plasticity

Despite the effects of PEMFs as still controversial, it has been shown that deep brain stimulation by PEMFs can modulate the activity of neurophysiological circuits producing clinical benefits in AD patients [15]. Recently, brain stimulations with PEMFs have been developed and applied for the treatment of neurological disorders: for instance, the stimulation known as TMS which interacts in a noninvasive way with the nervous system [17]. Cortical changes induced by electromagnetic waves have shown results in improving the neuronal plasticity [18]. Indeed, an excitability increase of the cerebral cortex may affect the phenomenon of LTP, which in turn would support the synaptic plasticity and promote the recovery of degenerated functions [64]. Under these preconditions, there is a growing interest in the application of PEMFs as a possible therapeutic approach in psychiatric and neurological disorders [16]. PEMFs could be used to restore the cognitive performance, for instance, in clinical trials on AD. Recently, the electromagnetic waves have been demonstrated to modulate the functions of the cytoskeleton and to promote the neuronal differentiation and the neurogenesis in the hippocampus in vivo through the upregulation of the Cav-1 channel, *β*-III-tubulin, MAP2, and the brain-derived neurotrophic factor (BDNF) [29]. The latter is widely expressed in the brain and contributes to a variety of neuronal processes affecting the neurodevelopment, the survival, and the maintenance of the homeostasis of the nervous system in elderly [27]. In the adult brain, BDNF plays a key role in the modulation of the synaptic plasticity and it is essential for the regulation of memory. For these reasons, obtained data support the hypothesis that the electromagnetic waves could improve the brain neuroplasticity also through the modulation of the expression of neurotrophic factors [64].

## 2. Materials and Methods

### 2.1. PBMC Isolation

Peripheral blood mononuclear cells (PBMCs) were obtained from peripheral blood of 13 AD patients by means centrifugation on a 1077-density gradient (Histopaque® 1077, Sigma-Aldrich, Inc.). The mononuclear fraction was recovered and resuspended at the concentration of 2.5 × 10^6^ cells/ml in a RPMI 1640 Medium supplemented with 10% bovine calf serum and 1% penicillin/streptomycin (Euroclone, Logan, UT). Cell vitality was assessed by trypan blue dye exclusion method; then, PBMCs were distributed in a 96-multiwell plate (Corning) with a density of 5 × 10^5^ cells/200 *μ*l medium/well and incubated at 37°C in a humidified atmosphere with 5% CO_2_. For each patient, 3 PBMC cultures were exposed to LF-PEMF for 3 different durations: 15, 30, and 60 min. Nonexposed (i.e., sham) control cultures were set up in parallel.

### 2.2. Electromagnetic Bioreactor and PBMC Exposure to LF-PEMF

The experimental setup of our electromagnetic bioreactor was based on two solenoids (i.e., air-cored coils) connected in series and powered by a pulse generator (BIOSTIM SPT Pulse Generator from IGEA, Carpi, Italy) [58]. The solenoids had a quasi-rectangular shape (length, 17 cm; width, 11.5 cm), and their planes were parallel with a distance of 10 cm. According to our mathematical model [65], this distance caused a stimulus characterized by a magnetic induction module of circa 3 mT. In addition, the magnetic induction field was perpendicular to the surface where the cells were seeded and grew; the signal frequency was equal to about 75 Hz.

### 2.3. RNA Extraction

Total RNA was extracted from untreated and LF-PEMF-treated cells using the RNeasy Mini kit (Qiagen GmbH, Hilden) according to the manufacturer's instructions. Total RNA obtained from the replicate cultures of each treatment was pooled, and the quality of RNA was assessed by determining the RNA integrity number (RIN) (TapeStation, Agilent Technologies). A quantitative RNA analysis was performed using a fluorimetric methods by means of the Qubit® platform (Invitrogen, Grand Island, NY, USA) using the Quant-iT RNA Assay (declared assay range between 5 and 100 ng; sample starting concentration between 250 pg/*μ*l and 100 ng/*μ*l): 2 μl of RNA was added to 198 *μ*l of the working solution obtained by mixing 1 *μ*l of Qubit™ RNA Reagent to 199 *μ*l of Qubit RNA Buffer. The quantitation was performed following the calibration of the instrument with the Quant-iT RNA standards (0 and 10 ng/ml).

### 2.4. Real-Time Reverse Transcription PCR (qRT-PCR)

Quantitative real-time reverse transcription PCR (qRT-PCR) was performed using cDNA obtained following the reverse transcription reaction with the miRCURY LNA™ Universal RT microRNA PCR kit: 4 *μ*l of total RNA (5 ng/*μ*l) was added to 4 *μ*l of 5x reaction buffer, 2 *μ*l of enzyme mix, 1 *μ*l of synthetic spike-in, and 9 *μ*l of nuclease-free water; and the reaction was performed using a thermocycler (Bio-Rad, MJ Mini) for one reaction cycle at 42°C for 60 min and 95°C for 5 min, and the reaction products were immediately cooled at 4°C.

To evaluate the miRNA expression, qRT-PCR reactions were performed using the Universal cDNA Synthesis and SYBR® Green Master Mix kits. Amplification was performed in a 10 *μ*l reaction mixture containing 4 *μ*l of 1 : 80 diluted cDNA, 5 *μ*l of SYBR Green Master Mix, and 1 *μ*l of specific LNA probe. miR-107 LNA probe (50| AGCAGCAUUGUACAGGGCUAUCA |72), miR-335-5p LNA probe (16| UCAAGAGCAAUAACGAAAAAUGU |38), and miR-26b-5p LNA probe (12| UUCAAGUAAUUCAGGAUAGGU |32) are provided by Exiqon using the following reaction conditions: a first step of 10 min at 95°C followed by 45 amplification cycles of 10 sec at 95°C and a final step at 60°C for 1 min. Small nuclear RNA U6 (snU6) was used to normalize the expression data of miRNAs, and every assay was performed in triplicates using the Eco Real-Time PCR Instrument (Illumina, San Diego, CA).

To evaluate the expression of mRNA of BACE1, a protein that is a target of miRNA 107, specific primers were designed using Primer-BLAST software (http://www.ncbi.nlm.nih.gov/tools/primer-blast): BACE1: f: 5′-GCAGGGCTACTACGTGGAGA-3′; r: 5′-GTATCCACCAGGATGTTGAGC-3′.

GAPDH (glyceraldehyde 3-phosphate dehydrogenase) was considered as endogenous control, and the following specific primers were used: f: 5′-CTGAGAATGGGAAGCTGGTCAT-3′; r: 5′-TGGTGCAGGATGCATTGCT-3′.

qRT-PCR was performed by the Eco Real-Time PCR Instrument (Illumina, San Diego, CA), and the results were analyzed by the comparative ct method (ΔΔct method using the software package of the Eco Real-Time PCR System for the calculus of the 2^−ΔΔct^ value [66].

Statistical analysis: from ct raw data of triplicate analysis, means and standard deviations were calculated and the statistical significance was analyzed by one-way ANOVA with post hoc LSD test (a *P* value smaller than 0.05 was considered as significant).

## 3. Results

This paper is intended to investigate the ability of LF-PEMF to modulate the expression of proteins involved in Alzheimer's disease. To this purpose, 3 different miRNAs were selected following a bioinformatics analysis in the specialized database miRTarBase. In addition, a PubMed search was performed for miRNAs and Alzheimer's disease. Two miRNAs (miR-335-5p and miR-26b-5p) were selected because of their involvement in brain signaling, in particular, in the glutamate uptake and in LTP. miR-335-5p is able to downregulate MAPK1 (mitogen-activated protein kinase 1) messenger translation. This gene encodes for a member of the MAP kinase family, also known as extracellular signal-regulated kinase (ERK), which acts as an integration point for multiple biochemical signals and is involved in a wide variety of cellular processes such as proliferation, differentiation, transcription, regulation, and development. ERK activity contributes to the synaptic plasticity; in fact, ERK cascade signals act with a regulatory role on the AMPA glutamate receptor (AMPAR), a non-NMDA type ionotropic transmembrane receptor for glutamate characterized by four types of subunits called GRIA (glutamate receptor ionotropic AMPA, 1–4) [67]. This particular receptor is involved in the fast synaptic transmission of the central nervous system, is activated by the artificial glutamate analog AMPA, and represents the most common receptor in the nervous system. It has been recently demonstrated [68] that AMPAR activation promotes the nonamyloidogenic APP processing and suppresses neuronal A*β* production. In this scenario, miR-335-5p is able to directly downregulate ERK which, in turn, regulates AMPAR which is involved in the first phase of LTP.

hsa-miR-26b-5p regulates the expression of a large number of genes, among which it is noteworthy, the carrier vGLUT_2_ (SLC17A6) involved in the promotion of the LTP. The same miRNA downregulates the kainate receptors.

In addition, miR-107 was considered because of previously reported studies [39] that observed a reduced expression of this miRNA in AD patients. This miRNA targets the messenger of BACE1 which is involved in the processing of APP toward the A*β* peptide: an increased expression of miR-107 would contrast the APP cleavage which results in a smaller deposition of A*β* plaques in the brain.

**Figure 1:**
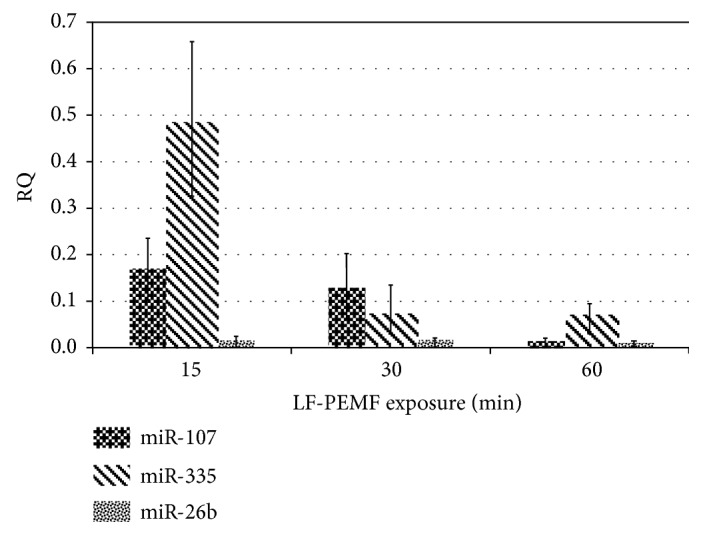
Expression of miR-107, miR-335, and miR-26b in PBMC from AD patients determined by relative quantification RQ (treated versus control sample). The values obtained after different times of exposure (15, 30, and 60 min) are shown (*P* > 0.05).

The ability of an electromagnetic field to modulate the expression of the selected miRNAs was tested on PBMC freshly isolated from the peripheral blood of 13 AD patients. The cells were exposed to LF-PEMF at 75 Hz for different durations (15, 30, and 60 min); subsequently, total RNA was extracted and cDNA was obtained as described in Section 2. The quantitative expressions of miR-107, miR-335-5p, and miR-26b-5p were determined by qRT-PCR using the small nucleolar RNA U6 as endogenous reference, and the RQ quantitative values were calculated against the untreated control cultures applying the ΔΔct method [66]. The results obtained are shown in Figure 1: mean data of 13 different PBMC cultures exposed for different times to LF-PEMF [3 mT; 75 Hz] are reported.

**Figure 2:**
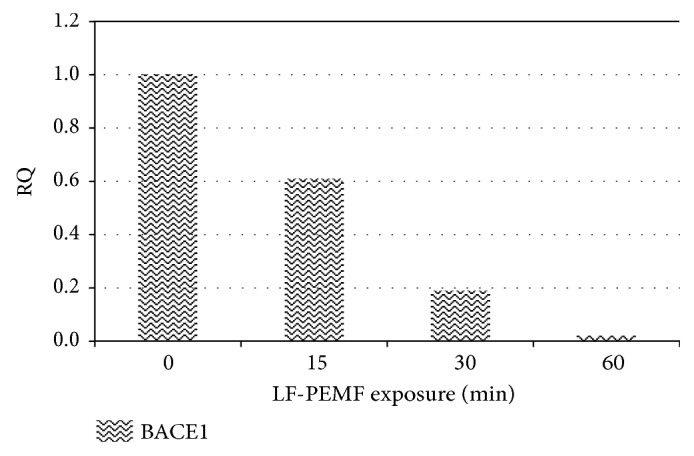
BACE1 expression in PBMC exposed to LF-PEMF for 3 different durations (15, 30, and 60 min). Relative quantification RQ of BACE1 mRNA using GAPDH mRNA as endogenous control (ΔΔct method [66]).

We can observe that the exposure to LF-PEMF was able to modulate the expression of all miRNAs considered; in particular, a progressive reduction of all miRNAs with the increasing time of exposure was observed even if the differences between untreated and treated cells were not statistically significant (*P* > 0.05). Similarly, the expression of BACE1 is affected by LF-PEMF with a progressive reduction of mRNA at the increasing exposure time (Figure 2).

**Figure 3:**
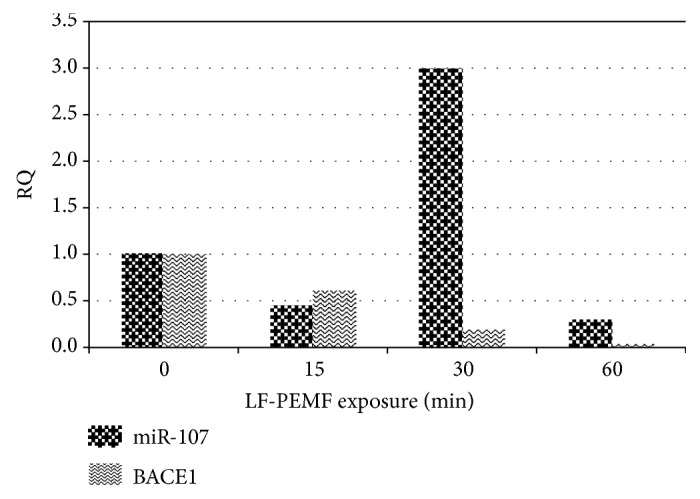
Comparison of miR-107 and BACE1 expression in the same PBMC culture exposed to LF-PEMF (15, 30, and 60 min durations). Results were normalized by U6 values for miR-107 and by GAPDH values for BACE1. Relative quantification RQ values were calculated against the untreated controls and referred to the average of the respective untreated control.

In Figure 3, the RQ values of both miR-107 and BACE1 mRNA obtained in one of the PBMC cultures, before and after LF-PEMF treatment with the different conditions, are compared. It can be observed that LF-PEMF induces a modulation of both miR-107 and of BACE1 mRNA expression. Moreover, a different modulation was observed depending on the duration of exposure.

## 4. Discussion

According to the present data, LF-PEMF (3 mT; 75 Hz) demonstrated to be able to modulate both miRNAs and mRNA involved in AD-related pathways.

miRNAs are molecules acting through direct complementary interaction with sequences of RNA messengers (target mRNA) and are able to interact with a broad range of mRNAs sharing the same sequences; so, each miRNA can be considered the center of a complex network that regulates various protein pathways. miR-107 has been seen to downregulate in addition to BACE1 and other mRNAs that could be involved in brain degenerative disorders, for example, GRN, CYP2C8, DAPK1, and PTEN. From literature data, diseases associated with GRN (granulin) include frontotemporal lobar degeneration with ubiquitin-positive inclusions and progressive nonfluent aphasia [69]. CYPP2C8 gene encodes a member of the cytochrome P450 superfamily of enzymes; these proteins are monooxygenases which catalyze many reactions involved in drug metabolism and synthesis of cholesterol, steroids, and other lipids. DAPK1 (death-associated protein kinase 1) is a gene responsible for atherosclerotic plaque development and destabilization. PTEN (phosphatase and tensin homolog) acts as a tumor suppressor downregulating AKT/PKB signaling pathway; moreover, it regulates intracellular levels of phosphatidylinositol-3,4,5-trisphosphate in cells. Another gene, which expression is regulated by miR-107, is SP1 encoding for a zinc finger transcription factor that binds GC-rich motifs of many promoters and is involved in many cellular processes, including cell differentiation, cell growth, apoptosis, immune responses, DNA repair, and chromatin remodeling.

Among the targets of miR-335-5p we consider particularly interesting tenascin C (TNC), an extracellular matrix protein implicated in the guidance of migrating neurons as well as axons during development, synaptic plasticity as well as neuronal regeneration; RASA1 (RAS P21 protein activator) that is an inhibitory regulator of the Ras/cyclic AMP pathway and stimulates the GTPase of normal but not oncogenic Ras p21; and IGFR1 (insulin-like growth factor 1 receptor), a transmembrane receptor that is activated by a hormone called insulin-like growth factor 1 (IGF-1) and by a IGF-1-related hormone called IGF-2. Another interesting target of miR-335-5p is APBB2 (A*β* precursor protein binding family B member 2) that encodes a protein interacting with the cytoplasmic domains of A*β* (A4) precursor protein and of A*β* (A4) precursor-like protein 2. The latter protein contains two phosphotyrosine-binding (PTB) domains, which are thought to function in signal transduction. In Table 1, some of the gene whose expression is regulated by the miRNAs studied are listed (source: miRTarBase, miRDB).

In conclusion, the results of the present study, using an ex vivo human PBMC model, demonstrated that LF-PEMF exposure really modulates the expression of miRNAs that regulate the brain signaling, so confirming the capacity of the electromagnetic field to stimulate both tissue regeneration and brain signaling. The analysis of changes in the expression levels of miRNAs, known as the regulatory processes involved in brain signaling and tissue regeneration, after LF-PEMF exposure, has allowed us to verify both the quantitative variations of these miRNAs and to identify other target messengers of the same miRNA. This has been possible through the analysis of protein networks in which the miRNAs are involved. In fact, each miRNA can interact through sequence complementarity with sequences contained in various target mRNAs and also can act in synergy with other miRNAs that regulate the same mRNA. The results of the present study confirmed the capacity of LF-PEMF to influence various networks of physiological functions that are dysregulated in AD. Among the effects observed, a quantitative reduction of *β*-secretase, following LF-PEMF exposure, could confirm a protective action of the electromagnetic field whose action would counteract the formation of A*β*. Expression values of miR-107 which is a negative regulator of BACE1 decrease with the increasing exposure time, and the same trend was observed for the expression of miR-26b-5p, which is involved in brain signaling and synaptic plasticity.

Differently, the expression of miR-335-5p, which negatively regulates the AMPA receptor, is stimulated by the electromagnetic field, even if this expression decreases with the increasing time of exposure. This result indicates a possible adverse effect depending on the time of exposure.

Overall, the results obtained from the study on our in vitro model demonstrated that LF-PEMF can stimulate an epigenetic regulation mediated by miRNAs, which would lead to a rebalancing of the pathways' deregulation occurring in AD (this deregulation starts in locus coeruleus and then continues in high-order association areas of the neocortex [70]). However, it is necessary to take account of the complex network of epigenetic signals, not yet completely known, and the possibility of some adverse effects. These results suggest that the electromagnetic fields at low frequencies, if properly used, may be useful for the treatment of patients with AD, as suggested by the results of pilot experiments with deep brain stimulation via EMFs, which were reported to produce clinical benefits [15]. However, for the complexity of the epigenetic regulation signals, which are triggered by electromagnetic stimulation [71–74], further in vitro and in vivo studies are needed in order to investigate the effects of LF-PEMF and in order to develop the conditions useful for a therapeutic use (e.g., via a dose-dependent epigenetic regulation mediated by miRNAs [75]), avoiding the possible adverse effects.

## Figures and Tables

**Table 1:** miRNAs studied and some of their targets that are involved in AD-related pathways.

ID	miRNA	Sequence	Target
MIMAT0000104	hsa-miR-107	50-agcagcauuguacagggcuauca-72	PLAG1, BACE1, CDK6, GRN, DAPK 1, PTEN, NOTCH 2, NFIA, SERBP1
MIMAT0000765	hsa-miR-335-5p	16-ucaagagcaauaacgaaaaaugu-38	TNC, RASA1, IGFR1, SP1, APBB2
MIMAT0000083	hsa-miR-26b-5p	12-uucaaguaauucaggauaggu-32	SLC17A6, (DNP1/vGLUT_2_)

*Note*. PLAG1: pleiomorphic adenoma gene 1; BACE1: beta-site APP-cleaving enzyme 1; CDK6: cyclin-dependent kinase 6; GRN: granulin; DAPK 1: death-associated protein kinase 1; PTEN: phosphatase and tensin homolog; NOTCH 2: Notch 2; NFIA: nuclear factor I/A; SERBP1: SERPINE1 mRNA-binding protein 1; TNC: tenascin C; RASA1: RAS p21 protein activator (GTPase-activating protein) 1; IGFR1: insulin-like growth factor 1 receptor; SP1: Sp1 transcription factor; APBB2: A*β* precursor protein binding family B member 2; SLC17A6 (DNP1/vGLUT_2_): solute carrier family 17 member 6 (vesicular glutamate transporter).
